# 

**Published:** 1892-11

**Authors:** 


Married.—The Journal acknowledges the courtesy of the following:

"Mr. and Mrs. J. E. Wallis invite you to the wedding reception of their daughter, Henry Pearl, and Dr. Robert W. Knox, on Thursday, November the tenth, eighteen hundred and ninety-two, at half past five o'clock. 1502 East Avenue I, Galveston, Texas."

The Journal extends hearty congratulations to the handsome young doctor.

